# Recent advances in renal interstitial fibrosis and tubular atrophy after kidney transplantation

**DOI:** 10.1186/1755-1536-7-15

**Published:** 2014-10-02

**Authors:** Xiaojun Li, Shougang Zhuang

**Affiliations:** 1 Department of Nephrology, Tongji University School of Medicine, Shanghai East Hospital, Shanghai, China; 2 Department of Medicine, Alpert Medical School of Brown University, Rhode Island Hospital, Middle House 301, 593 Eddy Street, Providence, RI 02903, USA

**Keywords:** Interstitial fibrosis, Renal transplantation, Renal allograft loss, Tubular atrophy

## Abstract

Although kidney transplantation has been an important means for the treatment of patients with end stage of renal disease, the long-term survival rate of the renal allograft remains a challenge. The cause of late renal allograft loss, once known as chronic allograft nephropathy, has been renamed “interstitial fibrosis and tubular atrophy” (IF/TA) to reflect the histologic pattern seen on biopsy. The mechanisms leading to IF/TA in the transplanted kidney include inflammation, activation of renal fibroblasts, and deposition of extracellular matrix proteins. Identifying the mediators and factors that trigger IF/TA may be useful in early diagnosis and development of novel therapeutic strategies for improving long-term renal allograft survival and patient outcomes. In this review, we highlight the recent advances in our understanding of IF/TA from three aspects: pathogenesis, diagnosis, and treatment.

## Review

For many years, chronic allograft nephropathy (CAN) was used to describe the progressive loss of renal function in transplanted kidneys over time not related to acute rejection. However, consensus began to form that the term did not sufficiently describe the underlying disease process. Interstitial fibrosis and tubular atrophy (IF/TA) describes the histologic characteristics of allograft destruction over time. While IF/TA has come to replace CAN [1], it is still not a specific disease, but a pattern of injury that has many underlying causes. The fundamental mechanism of interstitial fibrosis is the imbalance of extracellular matrix metabolism and abnormal accumulation via interaction of various inflammatory cytokines. Its pathogenesis has not been fully elucidated and existing therapy is not effective in improving renal transplant function.

### Pathogenesis of IF/TA

**Figure 1:**
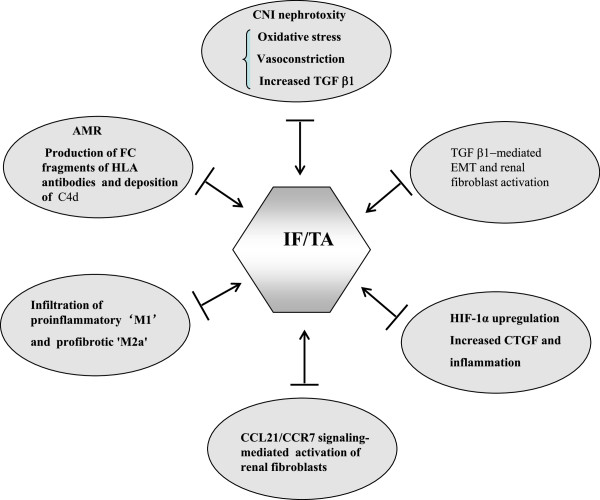
Identified mechanisms involved in IF/TA after kidney transplantation.

Previous studies indicated that IF/TA is a late feature of the renal allograft. However, increasing evidence has shown the same features of chronic histological damage as early as three months post-transplant. Moreover, the development of IF/TA is progressive, eventually resulting in chronic renal dysfunction [2]. IF/TA is associated with decreased graft survival, especially when it is accompanied by transplant vasculopathy, subclinical rejection, or transplant glomerulopathy. In a 3-month protocol biopsy study in which biopsies were classified according to the presence or absence of arterial intimal thickening, graft survival was significantly reduced in patients with transplant vasculopathy [3]. The simultaneous presence of IF/TA and incipient transplant glomerulopathy implies a shorter graft survival than the presence of IF/TA without transplant glomerulopathy. Moreover, it was reported that 10-year graft survival was 95% in patients with normal histology, 82% in patients with IF/TA without transplant vasculopathy, and 41% in patients with IF/TA and transplant vasculopathy [4]. In the past several decades, numerous studies have been conducted to understand the pathogenesis of IF/TA and multiple factors and mechanisms have been demonstrated to be involved in the progress of the IF/TA, including immunosuppressive drug toxicity, antibody-mediated injury, and epithelial–mesenchymal transition (EMT) (Figure 1).

### Immunosuppressive drug toxicity

Immunosuppressive drugs are closely associated with the development of IF/TA. Although the targets of immunosuppressive drugs are cells involved in the immune response, they also have toxic effects on epithelial, endothelial, and mesenchymal-origin cells [5]. Chronic nephrotoxic effects of calcineurin inhibitors may be associated with late allograft dysfunction and reduced allograft half-life. The introduction of calcineurin inhibitor (CNI) therapy – first cyclosporine (CsA) in the 1980s and later tacrolimus – was initially hampered by early dosing regimens that led to a wide variety of side effects. CNI can cause microvascular and glomerular damage, arteriolar hyaline deposition, tubular atrophy, and striped interstitial fibrosis. Nephrotoxicity in the first year post-transplant correlates with the 60% rate of such pathology in CNI-treated recipients [6].

The exact mechanism of immunosuppressive drug-mediated renal toxicity is not fully understood. It is evident that both cyclosporine and tacrolimus can cause renal and systemic vasoconstriction through increased release of endothelin-1, activation of the renin-angiotensin system, increased production of thromboxane A_2_, and decreased production of vasodilators such as nitric oxide and prostacyclin [7]. Cyclosporine can also cause oxidative stress through uncoupling mitochondrial oxidative phosphorylation, inhibition of the Krebs cycle, and activation of anaerobic glycolysis in the cytosol. In addition, tubulointerstitial fibrosis associated with CNI toxicity is also related to increased intrarenal transforming growth factor-β (TGF-β) mRNA expression [8]. TGF-β can promote interstitial fibrosis by decreasing the degradation and increasing the production of extracellular matrix proteins [9,10].

Activation of the mTOR pathway has been associated with extracellular matrix synthesis and renal fibrosis. It has been reported that blocking the mTOR pathway with rapamycin can reduce renal interstitial fibrosis in an obstructive nephropathy rodent model by diminishing the number of interstitial fibroblasts and myofibroblasts [11]. mTOR inhibitors also decrease TGF-β1 expression and significantly regress glomerular hypertrophy, mesangial fibrosis, and tubulointerstitial damage in various animal models of kidney injury and renal transplant patients [12,13]. Despite that inhibition of the mTOR pathway can attenuate renal fibrosis in animal models, application of rapamycin in patients with CNIs did not result in consistent beneficial effects. Pontrelli et al. [14] have reported that rapamycin can substantially reduce interstitial fibrosis in renal transplant recipients. Gonzalez et al. [15] demonstrated that switching from CNI to sirolimus for kidney transplants could also slow the course of IF/TA. However, Servais et al. [16] did not find a significant reduction in fibrosis after 1 year when patients were converted from CNIs to rapamycin 12 weeks after renal transplantation. Moreover, some recent studies have shown certain nephrotoxic potential of rapamycin especially when given in combination with high doses of CNIs [17,18].

### Antibody-mediated injury

IF/TA is the common pathological finding of various chronic kidney diseases including chronic renal allograft dysfunction resulting from antibody-mediated rejection (AMR), which is caused by circulating antibodies to donor alloantigens expressed on the endothelium. Several groups have recently reported that glomerulitis and peritubular capillaritis (microcirculation or microvascular inflammation) correlate with donor specific antibody (DSA) and graft failure in renal transplants [19-21]. There is also a wealth of literature indicating the incidence and deleterious impact of donor-specific HLA antibodies [22]. However, growing evidence suggests that anti-bodies against non-HLA antigens may also contribute to AMR in solid organ transplantation. Reports show that 10% to 23% of recipients are presensitized to non-HLA antigens [23,24], whereas 22% form non-HLA antibodies after transplantation [25]. The mechanisms of antibody-mediated graft injury are primarily driven by the effector functions of the Fc fragment of HLA antibodies, whereas experimental evidence indicates that the Fc promotes chronic inflammation and proliferation independent of antibodies [26-28].

Many studies have shown that C4d is an important marker of complement activation in the AMR, and C4d sediment can be found around renal tubular capillary by immunohistochemical staining techniques. Racusen et al. reported that, in biopsies of renal transplant patients suspected of having acute rejection, deposition of C4d complement fragments on the surface of endothelial cells is observed in more than 50% of peritubular capillaries and C4d3 is an important marker of an antibody-mediated immune response [29,30]. Recently, other studies have shown that C4d staining of glomeruli correlates with glomerulitis, an inflammatory lesion [31]. Valente et al. further pointed out that C4d staining of glomerular endothelial cells indicates glomerular endothelial damage [32]. However, C4d as a sign of AMR has certain limitations, because there is no expression of C4d in AMR. Sis et al. proposed that DSA titers have a higher sensitivity and accuracy than C4d in predicting progression to graft failure [19]. Renal transplant recipients with *de novo* DSA (dDSA) experience higher rates of rejection and worse graft survival than dDSA-free recipients. In a nested case–control study of adult kidney and kidney-pancreas recipients from July 2007 through July 2011 in a single center, Devos et al. demonstrated that development of dDSA is associated with increased incidence of renal graft loss [33] and graft failure after kidney transplantation [34]. Consequently, antibodies play an important role in the progression of renal allograft injury.

### The effect of macrophages on renal allograft injury

Previous studies indicated that macrophages exist within the transplanted kidney. These cells are derived from recruited monocytes. In addition to promotion or attenuation of inflammation and participation in innate and adaptive immune responses, macrophages mediate tissue injury and fibrosis, as well as tissue repair [35]. Recruited macrophages are generally divided into two phenotypes, M1 and M2, which have distinct functions. M1 phenotypes are proinflammatory macrophages that exacerbate renal cell damage, whereas M2 phenotypes are anti-inflammatory macrophages that promote epithelial and vascular repair. Insufficient vascular and epithelial healing despite abundant growth factor secretion would promote switch macrophages to profibrotic ‘M2a/wound healing’ macrophages that accelerate fibrogenesis and consequently renal allograft injury [36]. Evidence supports the notion that macrophages play an important role in promoting this process. For example, Qi et al. [37] have shown that macrophages mediate endothelial cell cytotoxicity leading to loss of renal microvasculature using a transgenic conditional ablation strategy to deplete circulating monocytes and infiltrating renal macrophages after kidney transplantation. Thus, it is evident that macrophage ablation reduced histologic features of rejection (arteritis, tubulitis) and the accompanying rarefaction of peritubular capillaries. The identification of macrophages immunopositive for inducible nitric oxide synthase implicated nitric oxide generation as a possible mechanism of endothelial cell cytotoxicity. These data indicate a significant role for macrophages in causing acute rejection-related tissue injury.

### Renal tubular epithelial to mesenchymal transition

IF is characterized by activation and proliferation of renal interstitial fibroblasts and accumulation of excessive amounts of extracellular matrix. The activation and expansion of matrix-producing cells occur through multiple sources and mechanisms, including activation of interstitial fibroblasts and pericytes, recruitment of circulating fibrocytes, and phenotypic conversion of tubular epithelial and endothelial cells [38,39]. EMThas been reported to contribute to the process of fibrosis in various organs, including kidney [40,41]. Several studies have shown that epithelial cells with an altered phenotype have been observed in transplanted kidneys with features of IF/TA [42]. Among the many fibrogenic factors that regulate renal fibrotic processes and EMT, TGF-β has been considered to play a central role [43-48]. TGF-β1 is upregulated in animal and human kidney allografts undergoing chronic rejection and chronic CsA-induced tubulointerstitial fibrosis [49,50]. TGF-β1 binding to the TGF receptor induces Smad2/3 phosphorylation. Smad2/3 are then translocated to the nuclei where they promote expression of TGF-β regulated genes including collagen I. In contrast, bone morphogenetic protein (BMP-7) has been identified as a natural antagonist of TGF-β1signaling and administration of exogenous BMP-7 also protects against renal fibrosis in several experimental models [51-54]. Furthermore, BMP-7 is effective in repressing expression of proinflammatory cytokines including interleukin-6 and interleukin-1, and chemokines in human renal tubular cells [55]. Thus, inhibition of EMT may improve clinical outcomes of renal transplant patients.

### Factors involved in inflammation and fibrosis of the renal allograft

#### A disintegrin and metalloproteinase 17 (ADAM17)

A disintegrin and metalloproteinase 17(ADAM17) is implicated in both pro-inflammatory and pro-fibrotic processes, which positions it as a possible target of intervention in a variety of diseases. It has been reported that an ADAM17 inhibitor was effective in reducing renal fibrosis in angiotensinII-induced kidney disease in mice [56]. Another study has also indicated that ADAM17-mediated production of soluble heparin binding epidermal growth factor (HB-EGF) is also involved in renal fibrosis via activation of EGF receptor (EGFR) signaling [57]. Therefore, ADAM17 may be implicated in interstitial renal damage after transplantation.

#### Hypoxia-inducible factor-1α (HIF-1α)

Studies have shown that infiltrating inflammatory cells are detected in IF/TA and contribute to long-term renal allograft failure [58,59]. For example, infiltrating monocytes/macrophages and their related chemokines/cytokines influence the long-term survival of renal allografts [60,61]. The infiltrating inflammatory cells contribute to IF/TA of chronic kidney transplant recipients through an HIF-1α signaling-dependent pathway. HIF-1αparticipates in fibrosis through regulating the expression of connective tissue growth factor (CTGF). Moreover, Yu et al. evaluated renal transplant recipients who underwent renal allograft biopsy with IF/TA, and found the expression of HIF-1α protein in filtrating inflammatory cells in areas with IF/TA in patients with chronic allograft dysfunction [62]. The expression of HIF-1α in the infiltrating macrophages/monocytes in chronic allograft dysfunction provides a novel role of HIF-1α in inflammation that may be caused by hypoxia which is not alloreactive [63]. HIF-1α may promote EMT development through regulating fibrotic gene expression during I/R injury in human renal tubular epithelial cells, and miR-21 could be among the important regulatory pathways in the process [64].

#### Fibroblast-specific protein chemokine CCL21 and chemokine receptor CCR7

The CCL21/CCR7 signaling pathway has been shown to participate in the development of renal fibrosis [65].It is well known that activation of fibroblasts is the key mechanism of kidney fibrosis [66]. Zhou et al. found that the CCL21/CCR7 signaling pathway contributes to renal allograft fibrosis through activation of renal fibroblasts. Furthermore, fibroblast surface protein-positive fibroblasts may be a risk factor for acute/active cellular rejection and chronic/sclerosing allograft nephropathy [67].

### Diagnosis

Early detection of IF/TA is important for effective management of potential chronically progressive injury in the transplanted kidney by minimizing risk factors associated with graft injury. At present, the gold standard is histological evaluation of tissue from renal biopsies. However, the currently used methods are ineffective, inaccurate, or invasive, and suffer from limitations in predicting outcomes. Recent studies have identified numerous specific biomarkers from blood and urine for monitoring of graft function after kidney transplantation that prove useful in earlier diagnosis (Table 1).

**Table 1:** Biomarkers in IF/TA after kidney transplantation

**Biomarkers**	**References**
**Blood**	
Malondialdehyde	[69]
Monocytes	[70–73]
MMP/TIMP system	[74-79]
DNA microchimerism	[80-87]
**Urine**	
mRNA : KIM-1	[91-94]
miRNA: miRNA-22, mir-140-3p, mir-125b, etc.	[95-97]
CCL2	[98-100]
CTGF	[101]
Vitamin D binding protein	[102]
Retinol binding protein	[103]

*MMP*, Matrix metalloproteinases; *TIMPs*, Tissue inhibitors of metalloproteinases; *KIM-1*, Kidney injury molecule-1; *CTGF*, Connective tissue growth factor.

### Blood biomarkers

#### Oxidative stress parameters

Oxidative stress may be the mechanism responsible for toxic effects and IF/TA caused by immunosuppressive drugs. It is evident that some immunosuppressive drugs, especially calcineurin inhibitors, contribute to an increase of oxidative stress [68]. Furthermore, oxidative stress is one of the most important components of ischemia/reperfusion process after kidney transplantation and increases with graft dysfunction. Fonseca et al. performed a prospective study of 40 renal transplantation recipients to evaluate time-dependent changes in oxidative stress-related parameters within the first week after transplantation and to assess their performance in predicting delayed graft function at one year. They found that increased malondialdehyde levels on day 1 after renal transplantation might be an early prognostic indicator of IF/TA, and levels on day 7 might represent a useful predictor of one-year graft function [69]. Therefore, monitoring oxidative stress will be beneficial to the early diagnosis of progression of IF/TA.

#### Monitoring circulating monocytes

Several studies have reported a relationship between excessive extracellular matrix protein and macrophage infiltrate. In addition, infiltrating macrophages correlated with formation of myofibroblasts. Increasing studies showed the accumulation of macrophages in damaged kidney allograft and macrophages are involved in the development and progression of kidney fibrosis. In animal models of chronic allograft nephropathy with IF/TA, macrophages are accumulated in the damaged kidney. In kidney recipients, the presence of macrophages in early biopsies is predictive of IF/TA [70,71]. Moreover, blockade of macrophage recruitment may reduce renal fibrosis [72]. Guillén-Gómez et al. also showed that monitoring monocytes could be a new tool for early identification of graft dysfunction in renal transplant patients by analyzing the phenotype of circulating monocytes [73]. However, the proposition also needs further experimental and clinical study.

#### Matrix metalloproteinases (MMPs)/tissue inhibitors of metalloproteinases (TIMPs) system

MMPs which belong to the large family of metzincins, are produced by renal cells (tubularepithelial cells, mesangial cells, and endothelial cells), and play a critical role in extracellular matirx remodeling [74,75]. However, MMPs can be specifically inhibited by tissue TIMPs. Increasing evidence reveals that the dysregulation of MMPs and TIMPs contributes to remodeling of kidney structure in patients with chronic allograft injury [76]. Recently, Mazanowska et al. proposed assessingTIMP-1 plasma levels to estimate allograft injury and suggested that they may be a useful biomarker in clinical practice to monitor for IF/TA [77]. In addition, serum MMP-2 and MMP-7 levels are higher in patients with IF/TA compared to kidney transplant patients with normal allograft function (estimated glomerular filtration rate (eGFR) ≥90 mL/min), suggesting potential non-invasive biomarkers for IF/TA [78]. Yanet al.have also reported that abnormal expressions of MMP-2 and TIMP-1 attributed to the development of IF/TA in chronic active antibody-mediated rejection [79]. Thus, monitoring the dysregulation of MMP/TIMP system may aid in the diagnosis of renal allograft fibrosis.

#### DNA microchimerism in blood of transplant recipients

The development of microchimerism, a phenomenon of the persistence of donor cells in the peripheral blood of renal transplant recipients, has been considered to be positively associated with the acceptance of transplanted organs [80,81]. Several case reports show that a microchimerism-positive finding in the recipients of renal transplantation is an index of acceptance of transplanted kidney, as shown by the relative longer survival time of transplanted kidneys in the recipients [82,83]. It was reported that the survival time of transplanted kidneys was significantly longer in microchimerism-positive recipients (8.7 years) than in microchimerism-negative recipients (5.4 years). The serum creatinine levels, measured at 1 year after transplantation, were significantly lower in the microchimerism-positive recipients than in the microchimerism-negative recipients [84]. Although the exact mechanisms by which microchimerisms formed remain largely unknown [85,86], the microchimerism was proposed to be derived from kidney cells, organ-contained leukocytes, or blood stem cells [87]. From a clinical point of view, microchimerisms might be one of several immunological mechanisms associated with long-term graft survival.

### Urine biomarkers

#### Urine mRNA and miRNA

Non-invasive, cost-effective biomarkers that allow frequent and accurate monitoring of graft function are needed in kidney transplantation [88,89]. As a biofluid, urine allows repeated and non-invasive collection, and its molecular composition highly reflects intrarenal events [90]. Many researchers assess mRNA levels of urinary pellets for the evaluation of chronic allograft dysfunction with IF/TA [91,92]. Kidney injury molecule-1 (KIM-1) is a protein present in toxic and ischemic acute renal injury and in chronic kidney diseases [93]. Nogareet al. suggested that quantification of KIM-1 mRNA in urinary sediment cells may be used as a non-invasive biomarker of fibrosis in kidney grafts with IF/TA [94].

Recently, microRNAs (miRNAs) have emerged as a biomarker for a variety of diseases. Several studies indicated that global miRNA expression changes are associated with IF/TA of kidney allografts [95,96]. Maluf et al. established miRNA signatures in urinary cell pellet samples from patients with and without biopsy-proven IF/TA using microarrays [97]; they identified a number of differentially expressed miRNAs in urinary cell pellets in patients histologically diagnosed upon renal biopsy as having IF/TA. Moreover, through the analysis of differentially expressed miRNAs in urinary cells, 22 miRNAs were found to be associated with IF/TA in patients [97]. Thus, urine mRNA and miRNAs may be potential biomarkers for monitoring allograft function and anticipating progression of IF/TA.

#### Urinary CCL2

Early non-invasive markers that identify patients at risk of renal allograft loss may stratify patients for more intensive monitoring or therapy. CCL2 is a CCR2 receptor chemokine that is a chemoattractant protein for monocytes/macrophages, T cells, and natural killer cells, and is generated by multiple cell lineages, including local tubular and glomerular epithelial cells as well as infiltrating monocytes/macrophages and lymphocytes [98,99]. In addition, Ho et al. have demonstrated, in a multicenter renal transplant cohort, that urinary CCL2 at 6 months is an independent predictor for the development of IF/TA at 24 months [100]. They also found that urinary CCL2:creatinine at 6 months is an independent predictor of death-censored renal allograft loss.

#### Urinary CTGF

CTGF has been considered as a biomarker of chronic renal allograft injury characterized by TA/IF. Shi et al. have demonstrated that urinary CTGF is an early predictor of TA/IF using a rat model. In an allogenic rat kidney transplant model, they found that typical morphological changes including TA/IF in allograft appeared at week 8 and became very severe at week 12 post-transplantation. In addition, CTGF expression in epithelium was up-regulated early and urinary CTGF was markedly elevated from week 4. Serum creatininein recipients was stable before week 8 but increased tremendously at week 12. Urinary CTGF increases earlier than the appearance of biochemical abnormalities and pathological changes. Thus, measurement of urinary CTGF may offer a potential non-invasive strategy to predict the early onset of chronic renal allograft injury [101].

#### Urinary vitamin D binding protein and retinol binding protein

Increased urinary protein excretion is common after renal transplantation and portends worse outcome. Mirković et al. investigated the value of urinary vitamin D binding protein excretion (uVDBP) as a tubulointerstitial inflammation and fibrosis marker in adriamycin rats, and tested whether uVDBP parallels renal damage and responds to therapy intensification in humans [102]. They propose that uVDBP may be a novel urinary biomarker of tubulointerstitial damage, independently of albuminuria. Prospectively designed studies are needed to validate these findings and confirm their relevance in the clinical setting. It has also been proposed that urinary excretion of retinol binding protein is a sensitive marker of allografts at risk. Amer et al. analyzed urine samples from 221 individuals one year after renal transplantation, showing that urinary retinol binding protein excretion is a sensitive marker of allograft fibrosis, which can predict long-term graft loss independent of histology and urinary albumin [103].

### Treatment

Development of IF/TA is a complex process that involves multiple factors and system interaction. Currently available treatments cannot effectively slow the progression of IF/TA and improve renal graft function. Some newly developed approaches may be beneficial for prolonging renal graft survival in the future. Those strategies include anti-EMT agents, antioxidant therapy, tubular epithelial cell repair, and mesenchymal stem cell therapy.

### Anti-EMT agents

EMT of renal tubular epithelial cells is thought to contribute to the progression of renal tubulointerstitial fibrosis. Antagonism of EMT could thus postpone and reverse renal interstitial fibrosis. Norcantharidin (NCTD) is a promising agent for inhibiting renal interstitial fibrosis [104]. Li et al. suggest that NCTD can antagonize tubular EMT by inhibiting the Smad pathway [105]; as such, NCTD treatment may preserve the normal epithelial phenotype and moderate tubular EMT.

Increasing evidence suggests that recombinant human erythropoietin (rHuEPO) protects neurons and cardiomyocytes from acute insults. Lee et al. investigated the protective effect of rHuEPO on cyclosporine-induced renal injury, suggesting that rHuEPO has a renoprotective effect against cyclosporine-induced chronic renal injury [106]. Park et al. have observed that recombinant human erythropoietin could inhibit the progression of renal fibrosis in mice with complete unilateral ureteral obstruction and the TGF-β1-inducedEMT in MDCK cells [107].

Kidney transplant recipients usually have low vitamin D levels, especially in the early post-transplantation period. Bienaimé et al. studied a prospective cohort of 634 kidney recipients who underwent transplantation at a single institution and found that low 25-hydroxyvitamin D concentration measured 3 months after transplantation is an independent risk factor for interstitial fibrosis progression and is associated with a lower eGFR one year after transplantation [108]. In mouse models of renal fibrosis, Ito et al. have also demonstrated that 25-hydroxyvitamin D treatment prevents renal fibrosis through the suppression of TGF-β-SMAD signal transduction [109]. Synthetic ligands of the vitamin D receptor that target the TGF-β-SMAD signaling pathway, which is known to regulate fibrosis-associated gene expression, ameliorated renal fibrosis in two different mouse models [110]. Thus, further investigation of vitamin D and related compounds for treatment of humans with chronic kidney fibrosis will be interesting.

Studies indicate that rapamycin has antiangiogenic and antiproliferative effects. Wu et al. have reported that rapamycin can significantly attenuate tubulointerstitial damage in a UUO-induced rat model of renal fibrosis, suggesting that rapamycin may have the potential to delay the progression of tubulointerstitial renal fibrosis [111]. In addition, Ko et al. have reported that sirolimus retards the development of chronic allograft dysfunction in a rat model [112]. By analyzing 20 renal transplant recipients who were treated with rapamycin, Özdemir et al. have also found that rapamycin-treated patients have a lower incidence of diffuse interstitial fibrosis [113]. Studies are underway to test whether using rapamycin as part of a calcineurin-sparing regimen actually affects long allograft function.

As TGF-β is involved in the pathogenesis of chronic rejection in kidney transplants [114] and contribute to development of EMT [115-118], TGF-β might be a key target for treating chronic rejection [119,120]. Guan et al. [121] have evaluated the efficacy of an anti-TGF-β monoclonal antibody in the prevention of chronic rejection of renal allografts. They demonstrated that administration of anti-TGF-β antibody successfully reduces the severity of chronic kidney transplant rejection in a rat model, suggesting the therapeutic potential for the anti-TGF-β antibody to prevent the chronic rejection of kidney transplants or prolong kidney transplant survival in patients.

BMP-7 is a natural TGF-β antagonist and has powerful renoprotective and anti-fibrotic effects [122-124]. It has been reported that administration of BMP-7 reduces glomerular and tubulointerstitial fibrosis in various experimental models of acute and chronic renal injury. Most of these studies have suggested that the principal target of BMP-7 in the kidney are renal epithelial cells. It protects against renal fibrosis through counteracting the profibrotic effects of TGF-β1 in glomerular mesangial cells and renal epithelial cells.

### Antioxidant therapy

Oxidative stress inhibition is likely to be involved in delaying the progression of renal interstitial fibrosis. Evidence indicates that alpha-lipoic acid (ALA) is a powerful antioxidant and exhibits a protective effect against renal injury. ALA also improves albuminuria and pathology in diabetes by reducing oxidative stress [125]. Wongmekiat et al. demonstrated that ALA supplementation attenuates renal interstitial fibrosis in rats with obstructive nephropathy [126]. Oxygen free radicals are important components involved in the pathophysiological processes observed during ischemia reperfusion. Sehirli et al. indicated that ALA reverses ischemia reperfusion-induced oxidant responses and improves microscopic damage and renal function [127].

### Syndecan-1

Syndecan-1, a heparan sulfate proteoglycan, has an important role in wound healing by binding several growth factors and cytokines. Clearly, repair of renal tubular damage is a crucial step in restoration of renal function upon transplantation. In addition, the balance between tubular epithelium functional repair and injury of chronic inflammation and fibrosis, is a dominant factor that determines renal allograft function in the long term [128]. Celie et al. have proposed that syndecan-1 plays an important role in tubular epithelial survival and repair in the renal allograft. Up-regulating the expression of syndecan-1 may help shifting the balance in the renal allograft towards functional restoration rather than IF/TA [129].

### Everolimus

Everolimus is an immunosuppressive macrolide. The initial clinical trials of everolimus were conducted in combination with standard-dose CsA, a regimen that demonstrates an equivalent efficacy to standard-dose CsA and mycophenolate mofetil with regards to the incidence of acute rejection. In several subsequent trials, the efficacy of everolimus has been evaluated via CNI minimization protocol, a strategy that usually maintains the efficacy and preserves renal function [130-132]. The safety and efficacy of different everolimus levels in combination with reduced-exposure CNI have also been confirmed [133,134]. In the CENTRAL pilot study, the conversion from CNI to everolimus overnight at week 7 after kidney transplantation showed a significant improvement in renal function at 6 months [135].

### Mesenchymal stem cell (MSC) therapy

Endogenous resident MSCs have been shown to play important roles in local repair in the kidney, including maintaining the endothelium stabilized. Cell therapies applied to solid organ transplantation have gained interest in the last years, and among them, MSC therapy has gained much attention.In addition to the regenerative properties of resident kidney MSCs, exogenously administered MSCs enhance the intrinsic reparative capabilities of the kidney. Numerous experimental models have demonstrated that MSCs attenuate alloimmune responses by suppression of allogeneic T-cell responses both *in vitro* and *in vivo*[136,137]. In addition, Franquesa et al. demonstrated a therapeutic effect of MSC in attenuating the progression of IF/TA. MSC injection results in an effective and long-term protection against kidney allografts [138]. Other studies have also shown that allogeneic MSC injection could decrease proteinuria and fibrosis in a 5/6 nephrectomy model [139,140]. Moreover, in a Col4A3 knock-out model of chronic allograft dysfunction, syngeneic MSCs are able to reduce interstitial fibrosis, while allogeneic MSCs do not ameliorate the progression of the disease [141]. A recent clinical trial showed the unexpected deleterious short-term effects of MSC therapy when given MSCs at the early stages after transplantation [142]. Thus, MSCs may be effective in preventing the progress of IF/TA. However, the exact mechanism and its safety require further clarification.

## Conclusions

Long-term renal allograft survival after kidney transplantation remains variable, depending on a host of factors. Understanding the mechanisms leading to the final common pathway of IF/TA in the transplanted kidney is important for early diagnosis and development of treatment strategies to prolong allograft life. Recent studies have suggested that immunosuppressive drug toxicity, antibody-mediated injury, EMT, pro-inflammatory, and pro-fibrosis factors are involved in the IF/TA. Multiple approaches, such as monitoring blood and urine samples, may be promising tools for early detection of IF/TA. Treatments for IF/TA, such as anti-EMT agents, antioxidant therapy, tubular epithelial repairing, and mesenchymal stem cell therapy, are under investigation. With further development of therapies to prevent or at least slow the progression of interstitial fibrosis and tubular atrophy, the improved long-term survival of renal transplants and delaying the return to dialysis will be hopeful.

## Abbreviations

ADAM17: A disintegrin and metalloproteinase 17; ALA: Alpha-lipoic acid; AMR: Antibody-mediated rejection; BMP-7: Bone morphogenetic protein 7; CAN: Chronic allograft nephropathy; CNI: Calcineurin inhibitor; CsA: Cyclosporine; CTGF: Connective tissue growth factor; dDSA: *de novo* DSA; DSA: Donor specific antibody; EGFR: EGF receptor; eGFR: Estimated glomerular filtration rate; EMT: Epithelial–mesenchymal transition; HB-EGF: Heparin binding epidermal growth factor; HIF-1α: Hypoxia-inducible factor-1α; IF/TA: Interstitial fibrosis and tubular atrophy; KIM-1: Kidney injury molecule-1; miRNAs: microRNAs; MMPs: Matrix metalloproteinases; MSC: Mesenchymal stem cell; NCTD: Norcantharidin; rHuEPO: Human erythropoietin; TGF-β: Transforming growth factor-β; TIMPs: Tissue inhibitors of metalloproteinases; uVDBP: Urinary vitamin D binding protein.

## Competing interests

The authors declare that they have no competing interests.

## Authors’ contributions

XL wrote and revised the manuscript; SZ wrote and edited the manuscript. Both authors read and approved the final manuscript.
